# *Anaplasma phagocytophilum* in horses and ticks in Tunisia

**DOI:** 10.1186/1756-3305-5-180

**Published:** 2012-08-30

**Authors:** Youmna M’ghirbi, Hèla Yaïch, Abderazek Ghorbel, Ali Bouattour

**Affiliations:** 1 Laboratoire d’épidémiologie et microbiologie vétérinaire, service d’entomologie médicale, Institut Pasteur de Tunis, Tunis, 1002, Tunisia; 2 Université Tunis El-Manar, Tunis, Tunisia; 3 Service pathologie médicale des équidés et des carnivores, Ecole nationale de medicine vétérinaire de Sidi Thabet, Tunis, 2002, Tunisia; 4 Université de la Manouba, Manouba, Tunisia

**Keywords:** *Anaplasma phagocytophilum*, Horses, Ticks, 16S rRNA gene, nPCR, IFA, Tunisia

## Abstract

**Background:**

* Anaplasma phagocytophilum *, the causative agent of granulocytic anaplasmosis, affects several species of wild and domesticated mammals, including horses. We used direct and indirect methods to compare and evaluate exposure to * A. phagocytophilum * in horses in northern Tunisia.

**Methods:**

Serum from 60 horses was tested by IFA for antibodies to * A. phagocytophilum *, and whole blood was tested for * A. phagocytophilum * 16S rRNA gene using a nested-PCR. To examine the risk of * A. phagocytophilum * transmission, 154 ticks that had been collected from horses were examined for the presence of * A. phagocytophilum * by nested-PCR targeting 16S rRNA gene.

**Results:**

This is the first time that *A. phagocytophilum* has been detected in horses in Tunisia, with an overall seroprevalence of 40/60 (67%). Six of the seroreactive samples (10%) had an IFA titer of 1:80, 14 (23%) of 1:160, 8 (13%) of 1:320 and 12 (20%) a titer 1 ≥ 640. The seroprevalence revealed no significant regional and sex differences. In contrast, a significant difference was observed between breeds. Eight (13%) of the horses were positive for *A. phagocytophilum* in the PCR, with no significant breed and age differences. *Hyalomma marginatum* was a predominant tick species (130/154)*,* and 3 were infected by *A. phagocytophilum* (a prevalence of 2.3%). The concordance rate of *A. phagocytophilum* detection between IFA and PCR had a *k* value of −0.07.

**Conclusions:**

The results presented in this study suggest that horses infested by ticks in Tunisia are exposed to *A. phagocytophilum*.

## Background

Three former species of granulocytic bacteria, * Ehrlichia phagocytophila  Ehrlichia equi * and the agent of human granulocytic ehrlichiosis, are considered as one and the same species and have been renamed as * Anaplasma phagocytophilum * following the reorganization of the * Anaplasmataceae * and * Rickettsiaceae * families in the order Rickettsiales [1]. * Anaplasma phagocytophilum *, etiologic agent of the formerly called equine granulocytic ehrlichiosis, is now reported as equine granulocytic anaplasmosis (EGA), and was first recognized as an equine disease in California [2]. The disease was subsequently found in other parts of the US and in Europe [3-6]. The most common symptoms include fever, depression, lack of appetite, leg swelling, reluctance to move and yellowish gums. * A. phagocytophilum * is maintained in a zoonotic cycle between infected mammalian host reservoirs and ticks, mainly (or perhaps only) those of the * Ixodes ricinus * and * I. persulcatus * complex [7].

In North Africa (Morocco, Algeria and Tunisia), *Ixodes ricinus* seems to be the main (or perhaps only) vector, although *Hyalomma detritum* might also play a role in transmitting * A. phagocytophilum * to cattle [8]. Moreover, *A. phagocytophilum* is likely to circulate in a variety of ticks as they feed on dogs or reptiles, as observed in Tunisia, South Africa and Ghana [9,10], although it is unknown whether they can transmit it.

To our knowledge, no information is available regarding the presence of *A. phagocytophilum* in horses and ticks in Tunisia. The present study was undertaken to further elucidate some epidemiological aspects of this vectorial disease. We investigated the exposure of horses in Tunisia to this bacterium by using an indirect fluorescent antibody (IFA) test to detect reactive antibodies against *A. phagocytophilum*, and molecular methods (nested polymerase chain reaction, nPCR) to detect the DNA. In addition, the latter assay was used to detect *A. phagocytophilum* in Ixodid ticks infesting horses.

## Methods

### Study areas and blood samples

Between April and June 2008, sixty horses were identified from 4 different localities all in a humid bioclimatic zone: Sejnane (n = 15; 37°03'29.85"N 9°14'20.80"E; altitude 200 m), Tabarka (n = 5; 36°57'26.51"N 8°45'03.95"E; altitude 700 m), Aïn Draham (n = 10; 36°46'48.97"N 8°41'13.73"E; altitude 800 m) and Ghardimaou (n = 30; 36°26'58.43"N 8°26'10.59"E, altitude 250 m). Within each region, horses were selected randomly based on availability due to previous contact to individual horse owners and clinical veterinary practices. Favorable to * Ixodes ricinus * tick, this bioclimatic zone is characterized by oak forests (* Quercus faginea * and * Q. suber *) with an undergrowth of ferns (predominantly * Pteridium aquilinum *) and tree heather (* Erica arborea *) [11]. Local fauna is a mixture of domestic (cattle, sheep, goats and horses) and wild animals, mainly lizards (*Psammodromus algirus*), wild boar and several species of birds (*Erithacus rubeculaTurdus merula*).

The age, sex and relative degree of tick infestation of each horse were recorded. All horses studied were born in Tunisia and have not traveled outside the country. Five milliliters of venal blood were collected from a jugular vein of each horse and an aliquot of 1 ml was placed in an EDTA-tube, for nPCR analysis. The rest of the collected blood samples were placed in coagulant-free tubes and allowed to clot at room temperature (25 °C). Samples were centrifuged at 2500 rpm for 15 min and sera removed and stored at −20 °C for the IFA tests. Our survey did involve sampling of a minimal number of horses according to internationally recognized guidelines with the approval of the Human and animal Ethics Committee of Pasteur Institute of Tunis. 

### Tick collection

Ticks were collected systematically from each horse, beginning at the head and neck region, then the pectoral, axillary and inguinal regions, finishing at the tail. The ticks were placed in a single container and preserved in 70% ethanol. Adult ticks were identified to the species level using a published taxonomic key [12].

### Serologic analysis

Samples were screened for IgG against *A. phagocytophilum* using a commercially available IFA assay from Fuller Laboratories (US). This IFA assay utilizes *A. phagocytophilum*-infected HL60 cells. All samples were tested at a 1:80 as starting dilution in phosphate-buffered saline solution (PBS), as per the manufacturer’s protocol. The resulting reactions were visualized using standard fluorescence microscopy (400×), where a positive reaction appears as sharply defined apple-green fluorescent inclusions (*A. phagocytophilum* morulae) in the cytoplasm of infected cells. A negative reaction appears as either red-counterstained cells or fluorescence unlike that seen in the positive control wells. Horse seropositive samples were then analyzed to determine the titration end point and the antibody titers were summarized into four different groups: 1:80, 1:160, 1:320, >1:640. A positive and a negative serum control, provided in the kit, were added to each slide.

### DNA extraction and nested-PCR

DNA was extracted from 200 μL of horse blood and unfed adult ticks, using Dneasy Tissue Kit (Qiagen Inc., Germany) as per the manufacturer’s instructions, eluted with 90 μL of AE buffer and then stored at −20 °C until further use. DNA yields were determined with a NanoDrop®ND-1000 Spectrophotometer (NanoDrop Technologies, DE, USA).

Genomic DNA of *A. phagocytophilum* was amplified by nested-PCR (nPCR) using the 16S subunit rDNA gene primers described by Massung *et al.*[13]. The first PCR amplification of *A. phagocytophilum* agent was performed in a 25 μL reaction mixture containing 3 μL of each DNA template with ge3a (5’-CACAATGCAAGTCGAACGGATTATTC-3’) and ge10r (5’-TTCCGTTAAGAAGGATCTAATCTCC-3’) to amplify 919 bp of the 16S rRNA gene of *A. phagocytophilum* complex. Reaction conditions were as follows: 0.2 μM each primer, 200 μM each deoxynucleoside triphosphate (Amersham Pharmacia biotech, UK), 1.25 U of *Taq* polymerase (Amersham Pharmacia biotech, UK) and 1 × Taq buffer (1.25 mM MgCl_2_). The reactions were performed on an automated DNA thermal cycler (Perkin-Elmer 2400, CA) under the following conditions: initial denaturation at 93 °C for 1 min, annealing at 55 °C for 5 min and extension at 72 °C for 30 s. The first PCR product from each sample was then diluted 1:10 with distilled water and 3 μL of the diluted solution were used as the template DNA for the second PCR in a final volume of 25 μL. Species-specific amplification primers ge9F (5’-AACGGATTATTCTTTATAGCTTGCT-3’) and ge2 (5’-GGCAGTATTAAAAGCAGCTCCAGG-3’) were used to amplify a 546 bp portion from the 5’ region of the 16S rRNA gene to identify *A. phagocytophilum*. The other PCR conditions were the same as in the first PCR. Positive and negative quality controls were also included with each experiment. DNA extracted from *A. phagocytophilum* (kindly provided by D. Postic, Pasteur Institute of Paris) was used as positive control.

### DNA sequencing and data analysis

To confirm the nPCR results, positive *A. phagocytophilum* amplified DNA was analyzed by sequencing using species-specific primers as described previously [13]. The PCR products were purified using the ExoSAP cleanup procedure (Amersham Biosciences). All nucleotide sequences were obtained using the Big Dye Terminator v.3.1 Cycle Sequencing Kit (Applied Biosystems) and the 3130 automated sequencer (Applied Biosystems). The sequences generated in this study have been deposited in the GenBank database under accession numbers JX121282-JX121291 (Table 1) and then edited and aligned using the software programs BioEdit [14] and ClustalW [15]. The BLAST program (http://www.ncbi.nlm.nih.gov/BLAST) was used to compare and analyze the data sequences.

**Table 1:** **Genetic variations detected in partial sequences of 16S rRNA gene of* Anaplasma phagocytophilum *detected in horses and ticks**

		**Nucleotide position**^**2**^													
**GenBank accession number**^1^	**Source of detection**	**11**	**23**	**28**	**73**	**118**	**130**	**234**	**246**	**249**	**276**	**284**	**287**	**302**	**397**
JN990106	Tick	C	T	G	G	G	C	T	T	A	A	T	C	**C**	A
JX121282	Tick	*	*	*	*	*	*	*	*	*	*	*	*	*	*
JX121283	Tick	*	*	*	*	*	*	*	**W**	**R**	*	*	*	*	*
JX121284	Horse	*	*	*	*	*	*	**C**	*	*	*	*	*	*	*
JX121285	Horse	*	*	*	*	**A**	*	*	*	*	*	*	*	*	*
JX121286	Horse	*	*	*	*	*	*	*	*	*	*	*	*	**T**	*
JX121287	Horse	**T**	*	*	*	*	*	*	*	*	**T**	*	*	*	*
JX121288	Horse	*	**C**	*	**R**	*	*	*	*	*	*	*	*	*	*
JX121289	Horse	*	*	*	*	*	*	*	*	*	*	*	*	*	*
JX121290	Horse	*	*	**A**	*	*	**T**	*	*	*	*	*	**T**	*	*
JX121291	Horse	*	*	*	*	*	*	*	*	*	*	**G**	*	*	**T**

^1^ The sequences of the genotypes obtained in this work [GenBank: JX121282-JX121291] were compared to the sequence previously reported in China [GenBank JN990106]. ^**2**^ Unique nucleotides are indicated with bold underlined letters. Conserved nucleotide positions are indicated with an asterisk.

### Statistical analysis

IFA test results were compared with nPCR findings; the two methods were assessed with a concordance test, and a *Cohel k* value was calculated [16]. Chi-square test, done with a commercially available statistical program (EpiInfo 6.01), was used. Observed differences were considered to be significant when the resulting p value was less than 0.05.

## Results

### Horse population and tick collection

During the study, 60 horses from humid bioclimatic zones were examined: 29 Arabian horses and 31 of the Barb breed. The horses were 12 years old on average (range of 2–28 years). Age was classified into four groups of 1–4 years, 5–10 years, 11–20 years and over 21 years. The adult ticks collected from the horses (n = 243) were classified as *Hyalomma marginatum* (144 males and 72 females), *H. excavatum* (17 males and 2 females) and *Rhipicephalus bursa* (4 males and 4 females). Only 11 of the 60 (18%) of the examined horses were infested by at least one of these tick species.

### Serological test

An indirect fluorescence assay showed that sera of 67% of the analysed horse population reacted with *A. phagocytophilum* antigen. Horses (n = 30) from Sejnane, Aïn Draham and Tabarka had a high rate of *A. phagocytophilum* antibodies (80%). In Ghardimaou, the seroprevalence was 53%. Titration endpoints for *A. phagocytophilum* ranged from 1:80 to 1 ≥ 640. Six of the positive samples (10%) had an IFA titer of 1:80, 14 (23%) of 1:160, 8 (13%) of 1:320 and 12 (20%) of 1 ≥ 640. *A. phagocytophilum* seroprevalence of horses revealed non-significant regional (*χ*2 = 4.8; ddl = 3; P > 0.05) and sex (*χ*2 = 0; P = 1) differences but a significant breed difference (*χ*2 = 16.59; P < 0.05). In fact, 40% of the Barb horses and 26.6% of the Arabian horses were positives.

### nPCR amplification

*A. phagocytophilum* DNA was detected by nested PCR in 8 blood samples among 60 tested horses (13%), including 2 horses from Sejnane, 4 from Ghardimaou and two from Aïn Draham. These 8 horses were between 7 and 21 years old and included the two different breeds. Breed (*χ*2 = 0.02; P = 0.89) and age (*χ*2 = 0.94; P = 0.3) differences were non-significant. Four of the 8 positive horses were also seropositive for *A. phagocytophilum*, but 4 horses infected with *A. phagocytophilum* were IFA negative. The concordance rate of *A. phagocytophilum* detection between IFA and nPCR was −0.07.

Only 154 unfed ticks (engorged specimens were discarded) were screened (130 *H. marginatum*, 16 *H. excavatum* and 8 *R. bursa*) for *A. phagocytophilum* DNA by nPCR. Bacterial DNA was present in three male *H. marginatum*, detected as a single band of the appropriate size by nPCR using primers specific for *A. phagocytophilum*.

### Sequencing data

Ten different variants in sequences of the partial 16S rRNA gene of *A. phagocytophilum* are known to occur [GenBank: JX121282-JX121291]. Only two sequences were obtained from tick-derived amplicons [GenBank: JX121282, JX121283]*.* The remaining DNA extracted from *H. marginatum* male ticks led to a weak positive signal in PCR and was not sequenced.

The two sequences for the tick-derived amplicons [GenBank: JX121282, JX121283] differed from each other by 2 nucleotide positions (Table 1). One sequence [GenBank: JX121282] was 100% similar to the GenBank JN990106 sequence derived from adult *Ixodes persulcatus* collected from grass in China [17]. The second sequence [GenBank: JX121283] was 99% identical to the sequence identified in ticks in China [GenBank: JN990106].

The partial sequences of the amplicons derived from the 8 positive horses were submitted to the GenBank database under accession numbers [GenBank: JX121284-JX121291] (Table 1). BLAST search of the horse derived sequences against GenBank revealed 99% similarity with *Anaplasma phagocytophilum* isolate NE-16S-2 [GenBank: JN990106] except one sequence [GenBank: JX121289] which is identical to JN990106 (Table 1). Sequences differed by 12 nucleotide positions (Table 1).

Alignment of the ten partial 16S rRNA gene sequences revealed 9 different sequence variants with 14 distinctions of nucleotide positions (Table 1). Two of these different strains revealed [GenBank: JX121283, JX121288] mixed profiles of *A. phagocytophilum* strains with superposition of SNPs on the same sequence (Table 1). SNPs were confirmed by sequencing twice the corresponding amplicons by the ge9F and ge2 primers.

## Discussion

*A. phagocytophilum,* the causative agent of granulocytic ehrlichiosis, affects several species of wild and domesticated mammals, including horses. Our investigation shows that *A. phagocytophilum* infects horses in Tunisia.

Of 60 horses screened by IFA, 40 were seroreactive with *A. phagocytophilum* antigen, showing that horses in Tunisia are exposed to *A. phagocytophilum*. The high rate of *A. phagocytophilum* antibody prevalence in healthy animals in the visited farms indicates that the horses may have developed subclinical *A. phagocytophilum* infection. *A. phagocytophilum* seroprevalence (67%) in the present study was higher than in previous surveys conducted in France (11.3%) [18], Spain (6.52%) [19], Italy (7.79, 16.89%, 17.03, 9%) [20-23], Sweden (16.7%) [24] and Denmark (22.3%) [25]. Other studies conducted in the US and Asia also revealed lower seroprevalence [26-29]. However, our result showed a significant seroprevalence difference between Arabian and Barb breeds. This could be attributed to the fact that the latter horses, used for several forest duties, were more exposed to ticks.

Serological methods are often used to diagnose anaplasmosis mainly in investigations involving large numbers of animals. But these tests cannot provide sufficient information for a diagnosis of the disease [30]. To overcome the limitation of the serological test, 16S rDNA based nested-PCR was used to obtain molecular evidence of *A. phagocytophilum* infection. In addition, partial sequencing of the detected 16S rRNA gene of *A. phagocytophilum* confirmed the nested-PCR results. Thus PCR-based molecular assays are powerful tools for diagnosing infectious diseases and for detecting bacterial agents in clinical cases and arthropod vector samples alike. These assays were instrumental in identifying *A. phagocytophilum*[1,31].

Using nested-PCR, among 60 analyzed animals, *A. phagocytophilum* DNA was detected in eight horses for the first time in Tunisia. The rate of PCR-positive horse samples (13%), drawn from the humid zone of Tunisia, is similar to that on the island of Sardinia (15%) [6] but is higher than previous surveys conducted in Central Italy (8%) [22].

The comparison of serological and molecular results shows that there is no concordance between the two tests used. A serologically positive test against a PCR-negative result could correspond to a past infection. Among PCR-*Anaplasma* positive horses (n = 8), only 4 had antibodies reactive with *A. phagocytophilum*. The remaining horses may have been sampled very early in the course of infection before antibody response had occurred or be related to a failure in responding to acute *Anaplasma* infection. In naturally infected horses, immunity persists for at least two years and does not appear to depend on latent infection or carrier status [32]. It has also been demonstrated that indirect fluorescent antibody titers to *A. phagocytophilum* persist for approximately 300 days after experimental infection [26] and animals tend to become serologically negative progressively, unless reinfections intervene.

Regarding the entomological survey, ticks infesting tested horses belong to three different species (*H. marginatumH. excavatum* and *R. bursa*). *H. marginatum* was the most abundant tick species in the collected samples. All of these three tick species are active in spring [11], which was when we did our sampling. This led us to hypothesize that *H. marginatum* can be involved in transmitting *A. phagocytophilum* to horses. Indeed, the PCR study of these specimens (n = 154) revealed the presence of *A. phagocytophilum* in three *H. marginatum* ticks, but does not of course prove that the species can transmit the infection. This low number of positive PCR results could depend on the vector competence of the tested tick species, the time of year, the geographical region and the limited bacteraemia of horses, which might significantly reduce tick infectivity. In previous studies we also detected *A. phagocytophilum* infections in dogs and in 3% of *Hyalomma detritum* and 1% of *Ixodes ricinus* ticks [8,9]. The latter tick species is considered to be the main vector of *A. phagocytophilum* in Europe. The absence of *I. ricinus* ticks on the horses that we examined could be attributed to the collection season, as this tick is active in Tunisia from September to March, that is, during autumn and winter [11].

However, *A. phagocytophilum* is spread widely in the northern hemisphere [33] and has been detected by PCR in *Ixodes ricinus* in almost all countries in Europe [34]. In Germany, the overall prevalence ranges from 1.0% to 4.5% [35,36]. The overall prevalence in Europe ranges from 0.25% in Scotland to 57.1% in Italy [37,38] with great variation in every country: from 0.4% to 15% in France [39,40], from 3% to 57% in Italy [38,41,42], from 0.25% to 2% in Scotland [37], 3% in Estonia [43], from 5.1% to 8.7% in Austria [44,45], from 0.5% to 2.2% in Switzerland [46-48], up to 40.5% in Denmark [49] and up to 14% in various Eastern European countries [50-54].

There have also been reports about the detection of *A. phagocytophilum* in *I. spinipalpis* and *I. dentatus* in the US [55,56]. In Asia, *A. phagocytophilum* has been detected in the hard ticks * I. persulcatus and I. ovatus *[57,58]. Apart from the genus *IxodesA. phagocytophilum* has also been detected in *Dermacentor variabilis* in California and *D. silvarum* in China [59,60].

Seeking IgG against *A. phagocytophilum* provides an excellent screening method to explore the circulation of the bacteria; PCR therefore remains a critical tool for a faster and earlier diagnosis. Positive serological testing and DNA detection by PCR are both evidence of *A. phagocytophilum* circulation in Tunisia. Even clinical anaplasmosis in horses probably remains under-diagnosed in Tunisia, as most horses recover spontaneously and clinical signs resemble those caused by infections by such other pathogens as *Borrelia burgdorferiBabesia caballiTheileria equi*, equine herpesvirus, equine infectious anaemia virus, equine arteritis virus and Leptospiraceae [61]. Similarly, an earlier study of *A. phagocytophilum* has indicated that up to 50% of seropositive horses, in endemic areas, have a subclinical infection [26]. It is assumed that clinical equine granulocytic anaplasmosis is an overlooked condition in most of Europe, as most horses recover spontaneously and clinicians do not examine them [62].

## Conclusions

This study marks the first report providing serological and molecular evidence of *A. phagocytophilum* infection in horses and ticks in Tunisia and should therefore be of interest to veterinary clinicians regarding the possible existence of clinical cases of equine anaplasmosis. Given the zoonotic aspect of *A. phagocytophilum*, physicians should also be informed of the presence of this bacterium in Tunisia. No serologic evidence of exposure to *A. phagocytophilum* has been reported in humans in Tunisia to date and this bacterium has not yet been isolated or detected by PCR in clinical cases in Tunisia. In addition, the results show that more than one variant of *A. phagocytophilum*, using partial 16S rRNA gene, seems to be involved in equine granulocytic anaplasmosis in Tunisia Furthermore, the zoonotic importance of *A. phagocytophilum* lends support to the need to increase the surveillance of the presence of this pathogen in Tunisia.

## Competing interests

The authors declare that they have no competing interests.

## Authors’ contributions

YM carried out the serological, molecular genetic studies, the sequence alignment, the statistical analysis and drafted the manuscript. HY carried out the serological assay. AG participated in the design of the study and sample horse collection. AB conceived of the study, and participated in its design and coordination, helped to draft the manuscript and sample horse collection. All authors read and approved the final manuscript.
